# Depression of long non-coding RNA SOX2 overlapping transcript attenuates lipopolysaccharide-induced injury in bronchial epithelial cells via miR-455-3p/phosphatase and tensin homolog axis and phosphatidylinositol 3-kinase/protein kinase B pathway

**DOI:** 10.1080/21655979.2022.2083820

**Published:** 2022-06-08

**Authors:** Chunhua Yi, Tijun Gu, Yongchang Li, Qian Zhang

**Affiliations:** a Department of Emergency, The Affiliated Changzhou No.2 People’s Hospital of Nanjing Medical University, Changzhou, Jiangsu, China; b Department of Critical Care Medicine, The Affiliated Changzhou No.2 People’s Hospital of Nanjing Medical University, Changzhou, Jiangsu, China; c Department of Respiratory and Critical Care Medicine, The Affiliated Changzhou No.2 People’s Hospital of Nanjing Medical University, Changzhou, Jiangsu, China

**Keywords:** SOX2-OT, human bronchial epithelial cells, inflammation cell apoptosis, miR-455-3p, PTEN-PI3K/AKT pathway

## Abstract

Airway inflammation is associated with various respiratory diseases, and previous research has confirmed that long non-coding RNAs (lncRNAs) play imperative roles in inflammatory responses. However, the function of lncRNA SOX2 overlapping transcript (SOX2-OT) in airway inflammation remains enigmatic. This study aimed to investigate the effects of SOX2-OT on lipopolysaccharide (LPS)–induced cell injury in human bronchial epithelial cells, BEAS-2B, and its potential mechanisms. The results showed increased cell apoptotic ratio, production of inflammatory cytokines, higher expression of adhesion molecules and activation of NF-κB in LPS–stimulated BEAS-2B cells. In LPS–stimulated BEAS-2B cells, SOX2-OT up-regulation and miR-455-3p down-regulation emerged simultaneously. SOX2-OT knockdown or miR-455-3p over-expression restrained LPS–induced inflammation and injury. SOX2-OT sponged to miR-455-3p and functioned as a ceRNA. In addition, phosphatase and tensin homolog (PTEN) served as an endogenous target of miR-455-3p to modulate the phosphatidylinositol 3-kinase/protein kinase B (PI3K/AKT) pathway and disturb the alleviated consequence of miR-455-3p over-expression on LPS–induced BEAS-2B cell inflammation and cell injury. Our data demonstrated that SOX2-OT plays a pivotal role in LPS–induced inflammation and injury in BEAS-2B cells and exerts its function through the miR-455-3p/PTEN axis and modulation of the PI3K/AKT pathway.

## GRAPHICAL ABSTRACT

**Figure:**
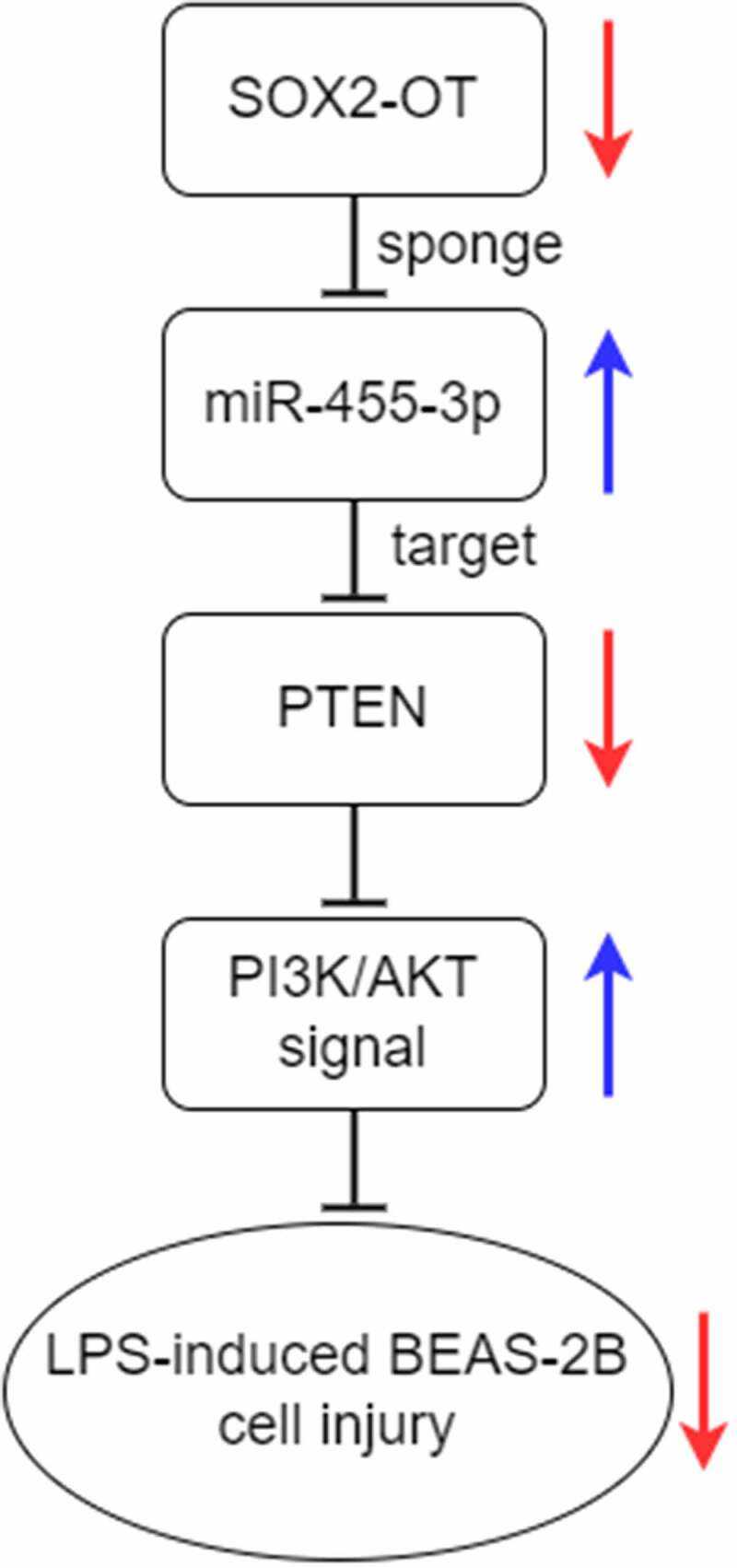

## Highlights

- SOX2-OT effects on LPS-induced cell injury in BEAS-2B cells were investigated.
- SOX2-OT knockdown/miR-455-3p over-expression inhibits LPS-induced inflammation.
- SOX2-OT sponged to miR-455-3p and functioned as a ceRNA.
- PTEN served as an endogenous target of miR-455-3p to modulate the PI3K/AKT pathway.
- SOX2-OT affects LPS-induced injury via miR-455-3p/PTEN and PI3K/AKT modulation.

## 1. Introduction

Airway inflammation is a significant and inevitable pathological process mediating diverse respiratory diseases, such as asthma, pneumonia, upper respiratory tract infections and chronic obstructive pulmonary disease [1]. For children and the elderly, respiratory diseases are common causes of mortality and morbidity [2]. The bronchial epithelium is the first anatomical barrier for stimulus and pathogens. Once a stimulus activates bronchial epithelial cells, a series of inflammatory cascades are initiated, accompanied by excessive release of inflammatory cytokines, which eventually lead to cell apoptosis [3,4]. To better understand this complex process, it is important to elucidate the regulatory mechanisms and identify more effective targets of inflammatory response in bronchial epithelial cells.

Long non-coding RNAs (lncRNAs) are RNA transcripts composed of longer than 200 nucleotides with insufficient protein-coding potential [5]. Recent research has shown that lncRNAs are drawn into multiple biologic processes, such as the regulation of innate immunity and inflammatory response [5–7]. SOX2 overlapping transcript (SOX2-OT), primarily located on the cytogenetic band 3q26.33, works as a transcriptional enhancer of SOX2 [8,9]. The SOX2-OT gene function has gained increased attention within the last five years. Previous studies have hinted that the SOX2-OT gene is connected to various human diseases, such as cancers, mental illnesses, heart diseases, and diabetic complications [9–11].

lncRNA SOX2-OT/miR-345-5p/epidermal growth factor receptor (EGFR) ceRNA network may explain the malignant transformation of human bronchial epithelial cells exposed to ambient particulate matter 2.5 [12]. Recent evidence indicated that inhibition of SOX2-OT enhanced inflammatory response and decreased apoptosis and oxidative damage in ischemic heart failure rate and oxygen and glucose deprivation H9C2 cell models through targeted revision of the miR-455-3p/TRAF6 axis [13]. However, the effect of SOX2-OT on lipopolysaccharide (LPS)-mediated cell injury in human bronchial epithelial cells has not been elucidated. This study investigated how SOX2-OT knockdown affects LPS-mediated BEAS-2B cell injury, such as apoptosis and inflammation. The findings might provide a novel insight into the treatment of respiratory diseases.

## 2. Materials and methods

### 2.1. Cell culture conditions and LPS treatment

Human bronchial epithelial cells BEAS-2B were acquired from American Type Culture Collection (Manassas, VA, USA) and grown in a bronchial epithelial cell medium (ScienCell, CA, USA) supplement with 10% Fetal bovine serum (FBS) (Hyclone, UT, USA) and 1% antibiotics (Invitrogen, CA, USA) at 37°C [14,15]. Cells in the logarithmic growth phase were trypsinized and seeded into 6-well plates, 35-mm culture dishes or 96-well plates according to the appropriate assay conditions. The cell injury model was established by stimulating with LPS (Sigma-Aldrich, MO, USA) at various concentrations (0, 0.25, 0.5, 1, and 2 μg/ml) for 24 h [14–17].

### 2.2. Cell transfection

To suppress SOX2-OT expression, antisense oligonucleotides, ASOs (small single-stranded nucleic acids that bind to SOX2-OT inside the cells), targeting SOX2-OT were designed and synthesized by RiboBio company (Guangzhou, China).

When BEAS-2B cells plated into 6-well plates or 24-well plates grew to 80% confluence, they were transfected with SOX2-OT-targeting ASO (ASO-SOX2OT), negative control ASO (ASO-NC), negative control miR (miR-NC), miR-455-3p mimic (miR-mimic), miR-455-3p inhibitor (miR-inhibitor), pcDNA3.1-control (pcDNA-Con) or pcDNA3.1-PTEN (Genepharma Co.Ltd., Shanghai, China) by Lipofectamine^TM^ 3000 reagent (Invitrogen) according to manufacturer’s instructions for 48 h [11–13]. After the transfection efficiency was verified by quantitative real-time PCR (qRT-PCR), the cells were collected and used for further analyses.

### 2.3. Total RNA isolation and qRT-PCR

According to the manufacturer’s protocol, total RNAs were isolated from BEAS-2B cells with Trizol kit (Invitrogen). Two micrograms of RNA was transcribed into cDNA, and the samples were subjected to qRT-PCR analysis with an SYBR Premix Ex TaqTM kit (Takara, Tokyo, Japan) or mirVana^TM^ qRT-PCR microRNA Detection kit (Ambion, TX, USA) [11–13]. The primers of PCR are listed in Supplemental Table S1. GAPDH and U6 were employed as internal references for SOX2-OT/phosphatase and tensin homolog (PTEN) and miR-455-3p. Target gene expression was quantified by the 2^−ΔΔCt^ method.

### 2.4. Enzyme-linked immunosorbent assay (ELISA) and lactate dehydrogenase (LDH) measurement

BEAS-2B cells were exposed to various treatments for the appropriate duration according to the scheduled experiments and centrifuged at 1200 × g for 10 min to gather the cell culture supernatants. The secretion of inflammatory cytokines and LDH analysis were determined by commercial ELISA kits (Abcam, Cambridge, MA, USA) and LDH Cytotoxicity Assay kit (Beyotime, Shanghai, China) following the protocols provided by the manufacturers [16,17]. All experiments were performed in triplicate.

### 2.5. Cell counting kit-8 (CCK-8) assay

Cell viability was determined by CCK-8 (Dojindo, Tokyo, Japan) [16]. BEAS-2B cells were treated as described above. CCK-8 reagent was added, and the cells were incubated for 2 h. An absorbance of 490 nm was measured with a microplate reader (Bio-Tek, VT, USA). All experiments were performed in triplicate.

### 2.6. Flow cytometry assay

FITC-Annexin V/PI Apoptosis Detection kit (Beyotime, Shanghai, China) was employed to assess cell apoptosis following the manufacturer’s protocol [16,17]. Briefly, treated cells were collected and dyed with Annexin V-FITC and PI for 15 min in the dark. The apoptotic cell ratios were evaluated with flow cytometry (Accuri C6, BD Biosciences, CA, USA).

### 2.7. Luciferase reporter assay

Predicted SOX2-OT or PTEN sequences containing miR-455-3p binding sites were inserted into the pmirGLO (Promega, WI, USA) and amplified by PCR to construct recombinants (Wild-type SOX2-OT 3ʹUTR (SOX2OT-WT)/SOX2-OT 3ʹUTR Mutant (SOX2OT-MUT) and Wild-type PTEN 3ʹUTR (PTEN-WT)/PTEN 3ʹUTR Mutant (PTEN-MUT)). Co-transfection was conducted with the Lipofectamine^TM^ 3000 reagent (Invitrogen) for 48 h. Luciferase activity was recognized using a luciferase activity assay kit (Promega, WI, USA), and the mean luciferase activity of cells was normalized to renilla luciferase activity [11–13].

### 2.8. RNA immunoprecipitation (RIP) assay

The endogenous binding potential of SOX2-OT and miR-455-3p was confirmed by RIP assay using a Magna RIP RNA-Binding Protein Immunoprecipitation kit (Millipore, Billerica, MA, USA) [11–13]. BEAS-2B cells were lysed with RIP lysis buffer supplemented with protease and RNase inhibitors for 1 h at 4°C. Subsequently, the supernatant of cellular lysates was harvested at 12000 × g for 10 min. A/G magnetic beads coated with antibodies of Argonaute 2 (Ago2) (Abcam) or IgG (negative control, Cell Signaling Technology, MA, USA) were added to the supernatant and incubated overnight at 4°C. After that, the beads were collected and used for isolating RNAs with phenol/chloroform/isoamyl alcohol mixtures (the ratio was 125:24:1). The isolated RNAs were subjected to analysis by qRT-PCR.

### 2.9. Western blot

Cell proteins were disintegrated using pre-cooled RIPA lysis buffer (Beyotime, Shanghai, China) and quantified by the BCA method [8,11,13–17]. Sixty micrograms of protein was subjected to 10% SDS-PAGE gel and transferred onto a PVDF membrane (Millipore). The membrane was blocked using 5% skim milk powder diluted with TBST at 37°C for 1 h and probed with primary antibodies of ICAM-1 (Santa Cruz), VCAM-I (Santa Cruz), phospho-NF-κB p65/NF-κB p65 (Biosciences), PTEN (Abcam), p-PI3K/PI3K (Abcam), p-AKT/AKT (Abcam) and GAPDH (Beyotime) at 4°C overnight and then incubated with horseradish peroxidase-conjugated secondary antibody (Beyotime) for an additional 1 h at room temperature. The protein expression level was visualized using a chemiluminescence detection reagent (Beyotime) and calculated by Image J software, with GAPDH as the internal reference.

### 2.10. Statistical analysis

GraphPad Prism 8 software (GraphPad Software Inc., CA, USA) was used to analyze all the experimental data. Each experiment was performed in triplicate, and data were expressed as mean ± SD. ANOVA was used to analyze differences in multiple groups, and a comparison between two groups was conducted using Student’s t-test. *P* < 0.05 was considered statistically significant.

## 3. Results

### 3.1. SOX2-OT knockdown mitigates LPS-induced cell injury in BEAS-2B cells

**Figure 1.**
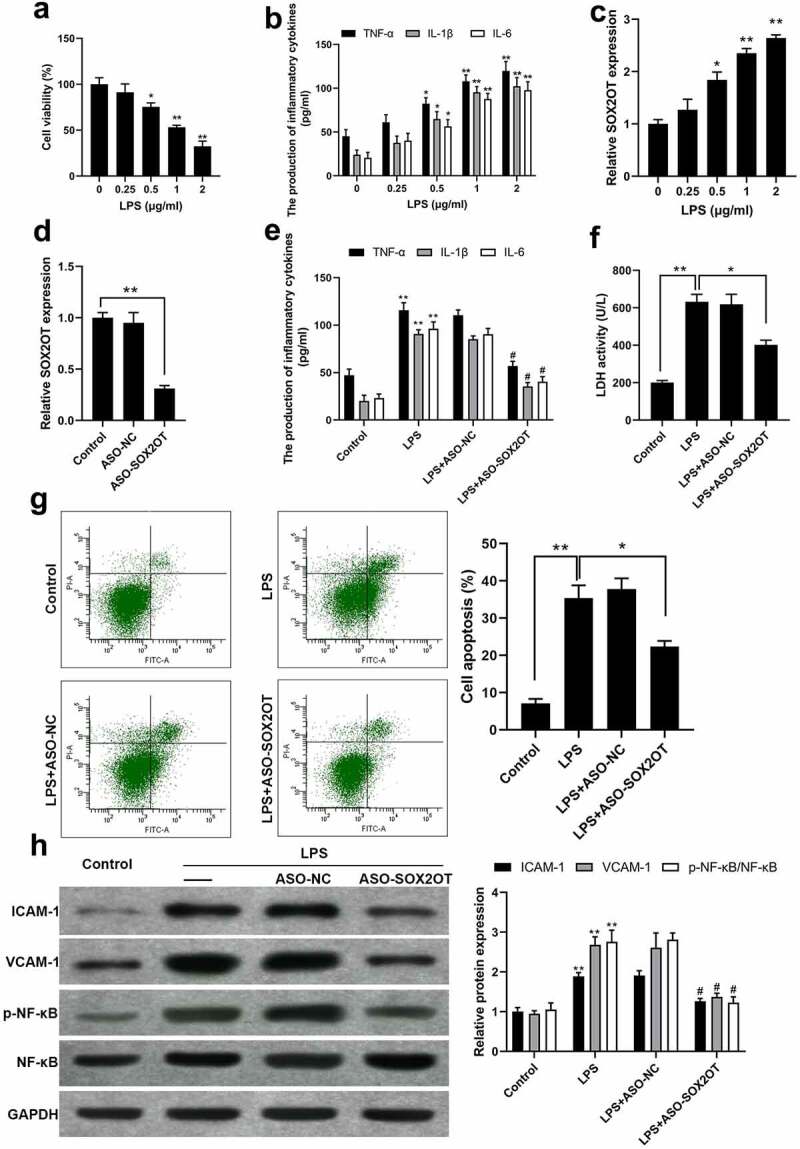
SOX2-OT knockdown suppressed inflammation and injury in LPS-treated BEAS-2B cells. (a-c) Cell viability (a), the production of inflammatory cytokines (b) and the expression of SOX2-OT (c) in BEAS-2B cells exposed to LPS at various concentrations; mean ± SD, n = 3, *P < 0.05, **P < 0.01 *vs* 0 μg/ml group. (d) Transfection efficiency of ASO-SOX2OT was confirmed by qRT-PCR; mean ± SD, n = 3, **P < 0.01. (e-h) Inflammatory cytokines levels (e), LDH activity (f), cellular apoptosis proportion (g), and protein expression levels (h) in 1 μg/ml LPS incubated BEAS-2B cells transfected with/without ASO-SOX2OT; mean ± SD, n = 3, **P < 0.01 *vs* Control group, ^#^*P* < 0.01 *vs* LPS+ASO-NC group.

We first examined whether LPS-induced cell injury in BEAS-2B cells was related to SOX2-OT expression. As presented in Figure 1(a,b), LPS treatment inhibited cell viability and enhanced the production of TNF-α, IL-1β and IL-6, indicating an inflammatory status. Interestingly, an increased expression of SOX2-OT was observed with the increase in LPS concentration (Figure 1(c)). These data suggest that BEAS-2B cell inflammation caused by LPS parallels a significantly increased SOX2-OT expression.

To determine the SOX2-OT function in LPS-induced BEAS-2B cell injury, SOX2-OT-targeting ASOs were transfected into LPS-induced cellular injury models. Transfection efficacy detection showed that ASO-SOX2OT remarkably reduced SOX2-OT expression (Figure 1(d)). ELISA assay (Figure 1(e)) revealed that ASO-SOX2OT transfection drastically lowered the LPS-induced production of TNF-α, IL-1β and IL-6 in BEAS-2B cells. Analysis of LDH showed that ASO-SOX2-OT decreased LPS-induced LDH activity (figure 1(f)). FITC-Annexin V/PI Apoptosis assay (Figure 1(g)) showed that SOX2-OT knockdown attenuated cell apoptosis of LPS-incubated BEAS-2B cells. Western blot assay (Figure 1(h)) demonstrated that SOX2-OT knockdown prevented the expression of ICAM-1, VCAM-1 and activation of NF-κB caused by LPS. These data indicate that SOX2-OT is up-regulated in LPS-incubated BEAS-2B cells and that SOX2-OT knockdown alleviates BEAS-2B cell injury prompted by LPS, such as inflammatory response, cell apoptosis, and activation of NF-κB.

### 3.2. The sponging relationship of SOX2-OT and miR-455-3p in BEAS-2B cells

**Figure 2.**
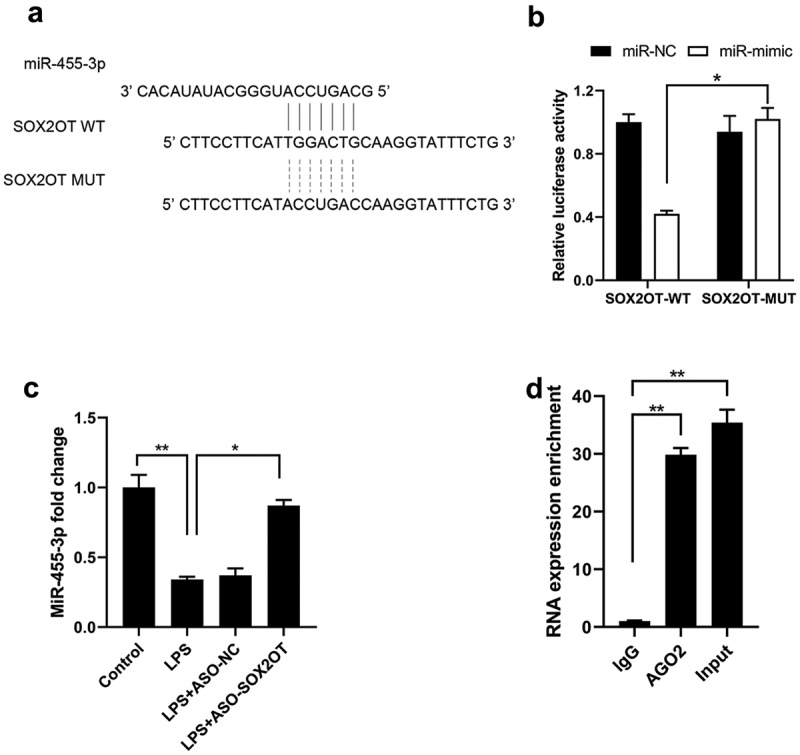
The sponging relationship of SOX2-OT and miR-455-3p in BEAS-2B cells. (a) Putative binding site of miR-455-3p on SOX2-OT; (b) Dual-luciferase activity of BEAS-2B cells cotransfected with miR-455-3p mimic and SOX2OT-WT/SOX2OT-MUT luciferase reporters; (c) The expression of miR-455-3p after SOX2-OT knockdown in LPS-treated BEAS-2B cells; (d) RIP assay assured that SOX2-OT interacted with miR-455-3p in BEAS-2B cells. mean ± SD, n = 3, *P < 0.05, **P < 0.01.

Evidence demonstrates that lncRNAs could function as ceRNA by competitively binding to miRNAs [5]. The bioinformatics websites miRDB and LNCipedia were used to predict the putative target miRNAs for SOX2-OT. As shown in Figure 2(a), miR-455-3p has a potential binding site with SOX2-OT. Luciferase reporter assay showed that in contrast to the miR-NC group, co-transfection of SOX2OT-WT and miR-455-3p mimic remarkably decreased the luciferase activity in BEAS-2B cells, while SOX2OT-MUT and miR-455-3p mimic co-transfection did not affect the luciferase activity (Figure 2(b)). An evident increase in miR-455-3p was also noticed in the ASO-SOX2OT transfection group in LPS-incubated BEAS-2B cells according to the qRT-PCR experiment (Figure 2(c)). The RIP results verified a positive correlation between SOX2-OT and miR-455-3p (Figure 2(d)). The abovementioned results demonstrate that SOX2-OT could sponge to miR-455-3p in BEAS-2B cells.

### 3.3. MiR-455-3p directly combined with PTEN and restrained the expression of PTEN

**Figure 3.**
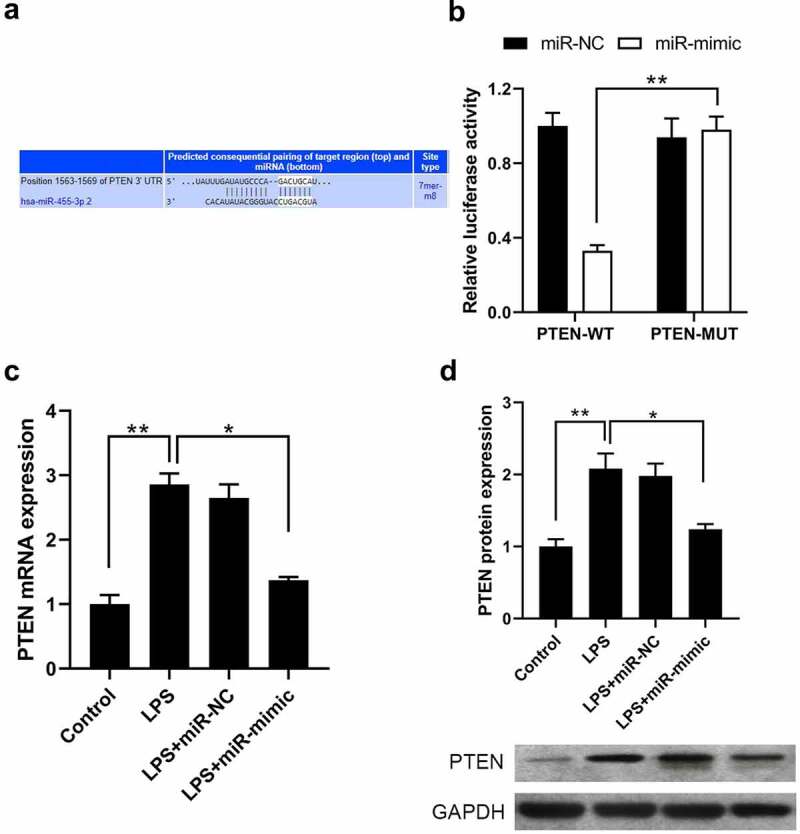
MiR-455-3p directly targeted PTEN in BEAS-2B cells. (a) Putative binding site of PTEN on miR-455-3p; (b) Dual-luciferase activity in BEAS-2B cells cotransfected with miR-455-3p mimic and PTEN-WT/PTEN-MUT luciferase reporters; (c) Detection of PTEN mRNA expression after transfection of miR-455-3p mimic in LPS-treated BEAS-2B cells; (d) Detection of PTEN protein expression after transfection of miR-455-3p mimic in LPS-incubated BEAS-2B cells. mean ± SD, n = 3, *P < 0.05, **P < 0.01.

We predicted potential target genes of miR-455-3p using TargetScan and recognized that PTEN was the best candidate and was selected for subsequent analyses (Figure 3(a)). To verify if miR-455-3p affects PTEN expression, luciferase plasmids of PTEN-WT and corresponding PTEN-MUT were constructed and cotransfected with miR-mimic/miR-NC. Results showed that miR-mimic repressed the PTEN-WT luciferase activity, not the mutant PTEN report vector (Figure 3(b)); this demonstrates that miR-455-3p targeted PTEN. We further examined PTEN expression after transfection with miR-mimic in LPS-incubated BEAS-2B cells. qRT-PCR and western blot analysis (Figures 3(c,d)) revealed that mRNA and protein levels of PTEN were down-regulated after transfection with the miR-455-3p mimic in LPS-treated BEAS-2B cells. These data suggest that miR-455-3p inhibits PTEN expression via targeting the binding 3’-UTR region of PTEN.

### 3.4. SOX2-OT depression suppressed LPS-induced BEAS-2B cells injury via miR-455-3p/PTEN axis

**Figure 4.**
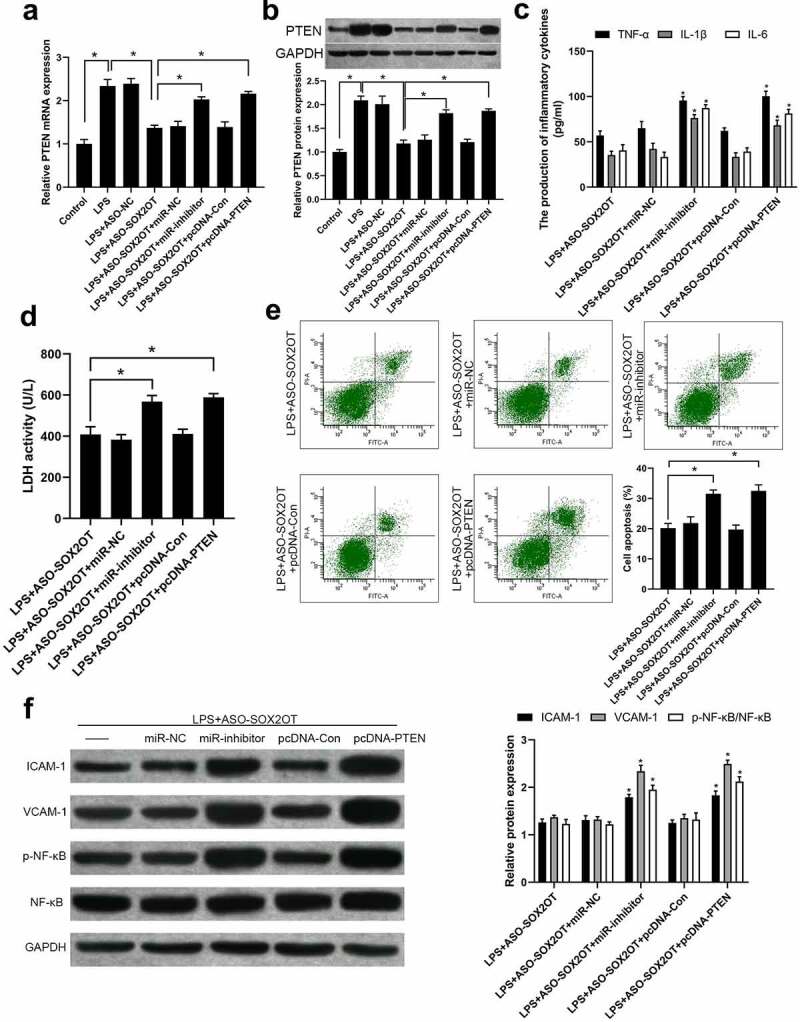
Knockdown of SOX2-OT restrained PTEN expression by enhancing miR-455-3p in LPS-incubated BEAS-2B cells. (a-b) The mRNA (a) and protein (b) level of PTEN expression in BEAS-2B cells transfected with different vectors; (c) TNF-α, IL-1β and IL-6 levels in each group; (d) LDH activity in each group; (e) Cellular apoptosis proportion in each group; (f) Proteins expressions in each group; mean ± SD, n = 3, *P < 0.05 *vs* LPS+ASO-SOX2OT group.

The inclusion of the miR-455-3p/PTEN axis in regulating the impact of SOX2-OT on LPS-induced cell injury was explored. Results showed that boost mRNA and protein expression of PTEN under LPS treatment was partly blocked by transfection of ASO-SOX2OT (Figure 4(a,b)). Notably, additional transfection with miR-inhibitor or pcDNA-PTEN could reverse the effect of ASO-SOX2OT on PTEN expression in LPS-treated BEAS-2B cells.

A series of rescue experiments were carried out. Of note, additional transfection of miR-455-3p inhibitor (LPS+ASO-SOX2OT+miR-inhibitor group) was associated with higher activity of LDH, higher secretion of TNF-α, IL-1β, IL-6, ICAM-1 and VCAM-1 than LPS+ASO-SOX2OT group, accompanied with augmented cell apoptotic ratio and activated NF-κB signal. Transfection of pcDNA-PTEN reversed the role of ASO-SOX2OT in LPS-treated BEAS-2B cells (Figure 4(c-f)). PI3K/AKT pathway activity was also evaluated; the data (Supplemental Figure S1) showed that LPS incubation reduced the phosphorylation of PI3K and AKT. Transfection of ASO-SOX2OT activated PI3K/AKT pathway in BEAS-2B cells. Additional transfection with miR-inhibitor or pcDNA-PTEN could avert the effect of ASO-SOX2OT.

## 4. Discussion

The inflammatory response of bronchial epithelial cells is a critical pathogenic mechanism of airway homeostasis and pulmonary diseases [1,3,4]. Considering the complications of airway inflammation, the emergence of novel targets might improve further understanding and treatment of chronic inflammation in the respiratory tract. During our report, special focus was given to SOX2-OT and its role, and mechanisms for regulating BEAS-2B cell injury were thoroughly explored. In the LPS-treated BEAS-2B cell injury model, SOX2-OT was up-regulated, while SOX2-OT depression weakened cell apoptosis, secretion of inflammatory cytokines and adhesion molecules, and inactivated NF-κB via miR-455-3p/PTEN axis. Based on our results, SOX2-OT should be considered a potential target in the treatment strategies for airway inflammation.

As the existing research confirmed the role of LPS in inflammation and related complications [18–20], we established the model of inflammation in *vitro* by exposing BEAS-2B cells to LPS. As airway epithelial cells are abnormally activated, there is excessive production of pro-inflammatory immune mediators, cytokines, and chemokines which eventually aggravate the inflammation [1,21–23]. Therefore, the inflammatory response was found in LPS-treated BEAS-2B cells accompanied by secretion of the pro-inflammatory cytokines TNF-α, IL-1β and IL-6 [24–26]. The elevation of SOX2-OT expression after exposure to LPS was found in our research. To inquire into SOX2-OT function, a SOX2-OT-targeting ASO was employed in this experiment, and the secretion of inflammatory cytokines was measured in BEAS-2B cells treated with LPS. The results showed that the increase in inflammatory cytokines production was rescued by knockdown of SOX2-OT. In addition, knockdown of SOX2-OT weakened the activation of LDH and cell apoptosis in LPS-treated BEAS-2B cells, which further proved that SOX2-OT knockdown has a protective effect on LPS-mediated bronchial epithelial cell injury. Considering the complex role of NF-κB in cell inflammation and apoptosis and that LPS elicits NF-κB activation [27,28], our experiments further clarified that SOX2-OT depilation decreased the phosphorylation of NF-κB. Besides mediating inflammation and cell apoptosis, the NF-κB signal also participates in the expression of ICAM-1 and VCAM-1, two typical adhesion molecules that may aggravate the inflammatory response and inflammatory cell recruitment [29,30]. In this study, SOX2-OT knockdown averted the expression of ICAM-1 and VCAM-1 induced by LPS in BEAS-2B cells.

Intriguingly, it is widely accepted that lncRNAs and miRNAs are associated to facilitate post-transcriptional regulation further [5]. Studies have revealed that SOX2-OT had prevalent interactions with a panel of miRNAs. For instance, SOX2-OT acts as a miR-627-3p sponge to promote Smads expression and is associated with the EGFR-TKIs resistance in NSCLC cell line H1975 [31]; in NSCLC cell lines A549 and H1299, SOX2-OT promotes cell migration and invasion by sponging to miR-132 [32]. Considering that the human SOX2-OT gene comprises multiple exons [33], its role in regulating LPS-evoked bronchial epithelial cell injury requires in-depth understanding. In this study, online prediction showed a binding potential of miR-455-3p and SOX2-OT and further verification experiments confirmed the negative correlation of miR-455-3p and SOX2-OT expression. In BEAS-2B cells, LPS inhibited miR-455-3p expression, while ASO-SOX2OT altered the inhibitory effect of LPS. The ameliorating effect of SOX2-OT knockdown on LPS-induced inflammatory cytokines secretion and cell apoptosis was partly reversed by miR-455-3p inhibitor. The molecular mechanism of SOX2-OT knockdown in alleviating LPS-induced cell injury in BEAS-2B cells could abrogate its suppression of miR-455-3p expression.

To better understand the molecular events, we also explored potential target genes of miR-455-3p in BEAS-2B cells. Our online prediction showed that PTEN was the best candidate gene for miR-455-3p. PTEN is involved in the pathogenesis of immune responses and plays a vital role in reversing serious cellular events, including cell growth, homeostasis, survival and metabolism [34–36]. Therefore, miR-455-3p can reduce the apoptosis of chondrocytes and alleviate osteoarthritis by targeting PTEN and regulating PI3K/AKT pathway [37]. In this report, we discovered that SOX2-OT knockdown could facilitate PTEN gene expression by negatively regulating miR-455-3p expression. Simultaneously replenishing miR-455-3p lessened LPS-induced PTEN expression in the mRNA and protein levels. Besides, previous studies confirmed that SOX2-OT was a regulator of PTEN [38] and PI3K/AKT signaling [39]. Decreased PTEN expression could activate PI3K signaling and amplify inflammation in COPD [40]. In our study, SOX2-OT cessation lowered PTEN expression and rescued the inactivated PI3K/AKT signal caused by LPS, which was altered by additional miR-455-3p inhibitor transfection. Rescue experiments showed that restoring PTEN nullified the suppressive effects of SOX2-OT knockdown on LPS-evoked cell apoptosis, NF-κB activation and production of inflammatory cytokines and adhesion molecules.

## 5. Conclusions

Collectively, our data demonstrate that SOX2-OT knockdown restrained LPS-induced BEAS-2B cell injury, such as inflammatory response, cell apoptosis, activation of NF-κB and expressions of ICAM-1 and VCAM-1. In addition, the mechanical analysis showed that SOX2-OT functioned as a ceRNA for miR-455-3p to modulate PTEN expression and PI3K/AKT signal, making it an attractive target for developing new treatment of airway inflammation.

## Supplementary Material

Supplemental Material
