# Supplementary material for: Depression of long non-coding RNA SOX2 overlapping transcript attenuates lipopolysaccharide-induced injury in bronchial epithelial cells via miR-455-3p/phosphatase and tensin homolog axis and phosphatidylinositol 3-kinase/protein kinase B pathway
Source: Bioengineered. 2022 Jun 8;13(5):13643–53. doi: 10.1080/21655979.2022.2083820 (PMC9275861; doi:10.1080/21655979.2022.2083820)
Supplement: Supplemental Material [file KBIE_A_2083820_SM9036.docx]

**Supplemental Table 1 The primers for qRT-PCR**

| Gene | Primer sequence |
| --- | --- |
| *SOX2-OT* | F: 5’-GTTCATGGCCTGGACTCTCC -3’ |
|  | R: 5’-ATTGCTAGCCCTCACACCTC -3’ |
| *miR-455-3p* | F: 5’-GCAGTCCATGGGCATATACAC-3’ |
| *PTEN* | F: 5’-TGGATTCGACTTAGACTTGACCT-3’ |
|  | R: 5’-GGTGGGTTATGGTCTTCAAAAGG -3’ |
| *GAPDH* | F:5’- CCACCCATGGCAAATTCCATGGCA-3’ |
|  | R: 5’- TCTAGACGGCAGGTCAGGTCCACC-3’ |
| *U6* | F: 5’-GTGCTCGCTTCGGCAGCACAT-3’ |
|  | R: 5’-ATGGAACGCTTCACGAATTTG-3’ |

**
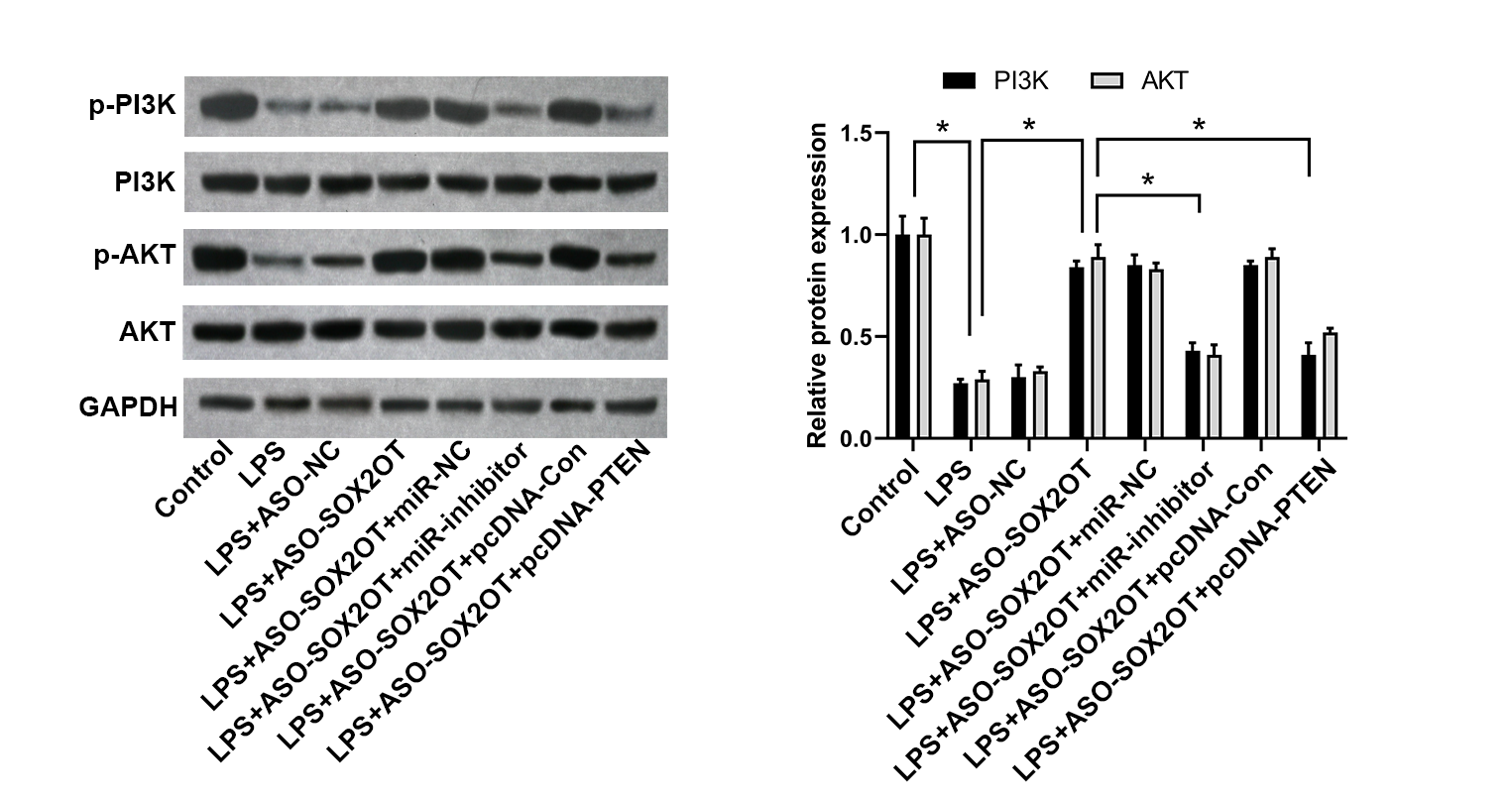
**

**Supplemental Figure 1. Knockdown of SOX2-OT modulatied activation of PI3K/AKT pathway in BEAS-2B cells.** The phosphorylation of PI3K and AKT assessed with western blot; mean±SD, n=3, **P*<0.05.
